# Transcatheter rectal arterial chemoembolization with oxaliplatin plus S-1 concurrent chemoradiotherapy can improve the pathological remission rate in locally advanced rectal cancer: a comparative study

**DOI:** 10.1186/s13014-020-01540-4

**Published:** 2020-05-06

**Authors:** Bo Yang, Jinlu Shan, Yan Feng, Nan Dai, Mengxia Li, Chuan Chen, Shengyong He, Ge Wang, Hualiang Xiao, Chunxue Li, Dong Wang

**Affiliations:** 1 Cancer Center, Daping Hospital & Army Medical Center of PLA, Third Military Medical University (Army Medical University), Chongqing, 400042 China; 2 Department of Pathology, Daping Hospital & Army Medical Center of PLA, Third Military Medical University (Army Medical University), Chongqing, 400042 China; 3 Department of General Surgery, Colorectal Division, Daping Hospital & Army Medical Center of PLA, Third Military Medical University (Army Medical University), Chongqing, 400042 China

**Keywords:** Rectal cancer, Chemoembolization, S-1, Neoadjuvant chemoradiotherapy, Clinical study

## Abstract

**Background:**

To explore the efficacy and safety of Transcatheter rectal arterial chemoembolization with oxaliplatin and S-1 concurrent chemoradiotherapy as neoadjuvant therapy for locally advanced rectal cancer.

**Methods:**

This s a prospective, monocentric, non-randomized clinical study, a total of 95 patients were enrolled and assigned to two groups: an investigational group (*n* = 50) receiving transcatheter rectal arterial chemoembolization (TRACE) with oxaliplatin and preoperative radiotherapy plus S-1 concurrent chemotherapy (NATRACE-CRT), followed by surgery, a control group (*n* = 45) receiving standard fluorouracil-based combined modality treatment, consisting of preoperative radiotherapy plus capecitabine based chemotherapy (NA-CRT), followed by surgery. The primary endpoint was postoperative pathological regression rate which evaluated by tumor regression grade (TRG) according to the 7th edition of the American Joint Committee on Cancer (AJCC) standard, and the secondary endpoints included objective response rate (ORR) and toxicity, as well as surgical complications, and postoperative tumor downstaging.

**Results:**

Compared with NA-CRT group (17.78% (95% confidence interval (CI): 6.2–29.4)), the TRG0 was 30% (95% CI 16.8–43.2) in the NATRACE-CRT group (*P* = 0.231). The TRG0 + 1 rate was 60% (95% CI: 45.9–74.1) and 33.33% (95% CI: 19–47.7) in NATRACE-CRT group and NA-CRT group, respectively (*P* = 0.013). The ORR of the NATRACE-CRT group was 84% and that of the NA-CRT group was 66.67% (*p* = 0.058). Incidence of preoperative toxic side effects and surgical complications was similar between the two groups.

**Conclusion:**

TRACE with oxaliplatin plus concurrent S-1 chemoradiotherapy as a neoadjuvant therapy provided better pathological remission rate versus standard treatment with a similar safety profile.

**Trial registration:**

NCT03601156.

## Background

The incidence and mortality of colorectal cancer are gradually increasing in China [1]. China’s cancer statistics showed that the ranks of incidence and mortality of colorectal cancer were the third and the second among all malignant tumors, respectively, of which rectal cancer accounted for about 28% [2]. Combined-modality therapy consisting of preoperative chemoradiotherapy (CRT), total mesorectal excision (TME) surgery, and postoperative chemotherapy have become standard treatment models for locally advanced rectal cancer (LARC) [3, 4]. Neoadjuvant chemoradiotherapy can improve the rate of local control and organ preservation, while pCR (pathologic complete remission) only can be achieved in a small number of patients [5, 6]. The majority of studies have revealed that the pCR rate after neoadjuvant therapy is about 10–25% [7], which has been hovering at a low level, regardless of the change of radiotherapy mode or chemotherapy regimen [8].

It has been reported that pCR, as a therapeutic response of neoadjuvant radiation response, is an independent predictor of disease-free survival (DFS) and overall survival (OS) [9]. Studies have shown that the 5-year survival rate of pCR patients is 92.6% and that of non-pCR patients is only 73.1% [10].

Transcatheter rectal arterial chemoembolization (TRACE) aims to control the tumor by injecting a single or multiple chemotherapeutic agents after the selective catheterization of the tumor feeding artery, thereby embolizing the tumor feeding artery [11]. At present, TRACE has been extensively used in the treatment of primary liver carcinoma and metastatic liver tumor [12], Roberto Bini runed a prospective mono-institutional study of TRACE-Debiri as an exclusive treatment for locally advanced rectal cancer not suitable for any further treatment option, and they found TRACE with Debiri could be a possible option for locally advanced/inoperable or recurred rectal cancer patients [13].

S-1 combines tegafur, gimeracil, and oteracil potassium in a molar ratio of 1:0.4:1. In JFMC35-C1: ACTS-RC trial, the researchers compared the efficacy of S-1 and tegafur–uracil in adjuvant therapy after curative surgery in LARC. The results showed that S-1 was superior to tegafur–uracil in 5-year RFS, with an increase of 4.7% in 5-year RFS, but there was no significant difference in 5-year OS between the two groups, and the side effects of the two groups were similar [14]. In addition, JACCRO CC-04: SHOGUN trial has been found that S-1, as neoadjuvant chemotherapy in LARC, can improve the pCR rate [15].

To explore the efficacy and safety of TRACE with oxaliplatin and S-1 concurrent chemoradiotherapy as neoadjuvant therapy for locally advanced rectal cancer. We designed this study and compared the efficacy and safety of this treatment regimen and 5-FU-based neoadjuvant chemoradiotherapy for LARC.

## Methods

### Patients

This is a prospective, non-randomized clinical study, and that was approved by the Ethics Committee of our university (Clinical Trial No. NCT03601156). Each patient signed the written informed consent form prior to start of the study.

Inclusion criteria were as follows: (1) age, 18–75 years old; (2) pathological diagnosis of rectal adenocarcinoma by rectosigmoidoscopy; (3) clinical diagnosis (magnetic resonance imaging (MRI)) of T3–4, any N; (4) distance between the tumor and anal margin < 12 cm; (5) Eastern Cooperative Oncology Group (ECOG) score of performance status≤1. Exclusion criteria were as follows: (1) distant metastasis was found by preoperative examination; (2) with other serious complications cannot complete treatment regimen, such as a surgical contraindication; (3) patients with a history of radiotherapy or chemotherapy.

**Fig. 1:**
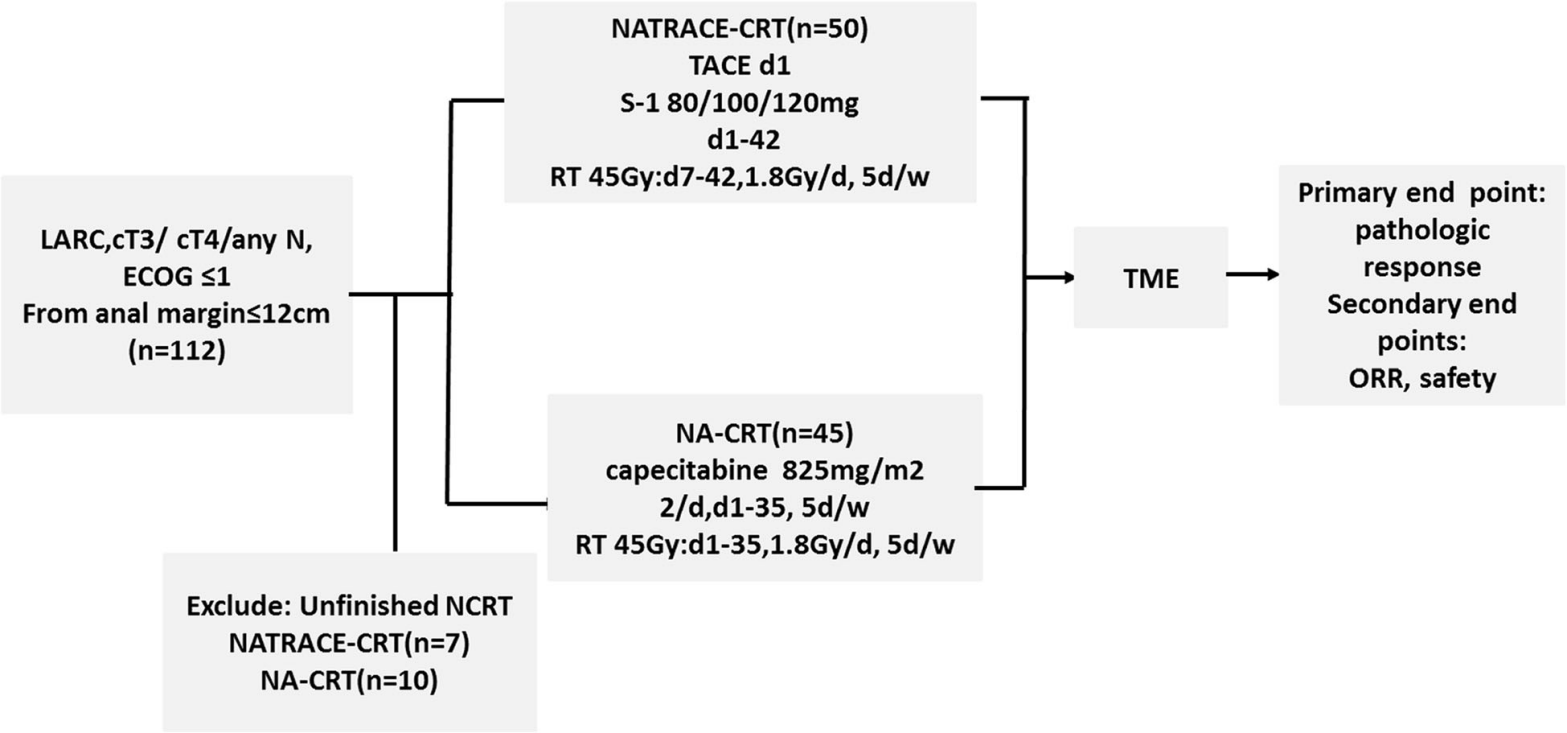
The study diagram. After enrollment, 17 patients were not included in the final analysis due to the absence of surgery (8) and incomplete chemoradiotherapy (9) respectively

According to the inclusion and exclusion criteria, 112 patients were recruited from our hospital from August 2015 to August 2018. Seventeen patients who did not complete NCRT and operation as planned were removed (7 in NATRACE-CRT group, 10 in NA-CRT group), Finally, 95 patients were divided into NATRACE-CRT group and NA-CRT group according to patient wishes, including 50 cases in the NATRACE-CRT group and 45 patients in the NA-CRT group (Fig. 1 shows the patients’ recruitment and treatment schema).

### Treatment regimen

**Fig. 2:**
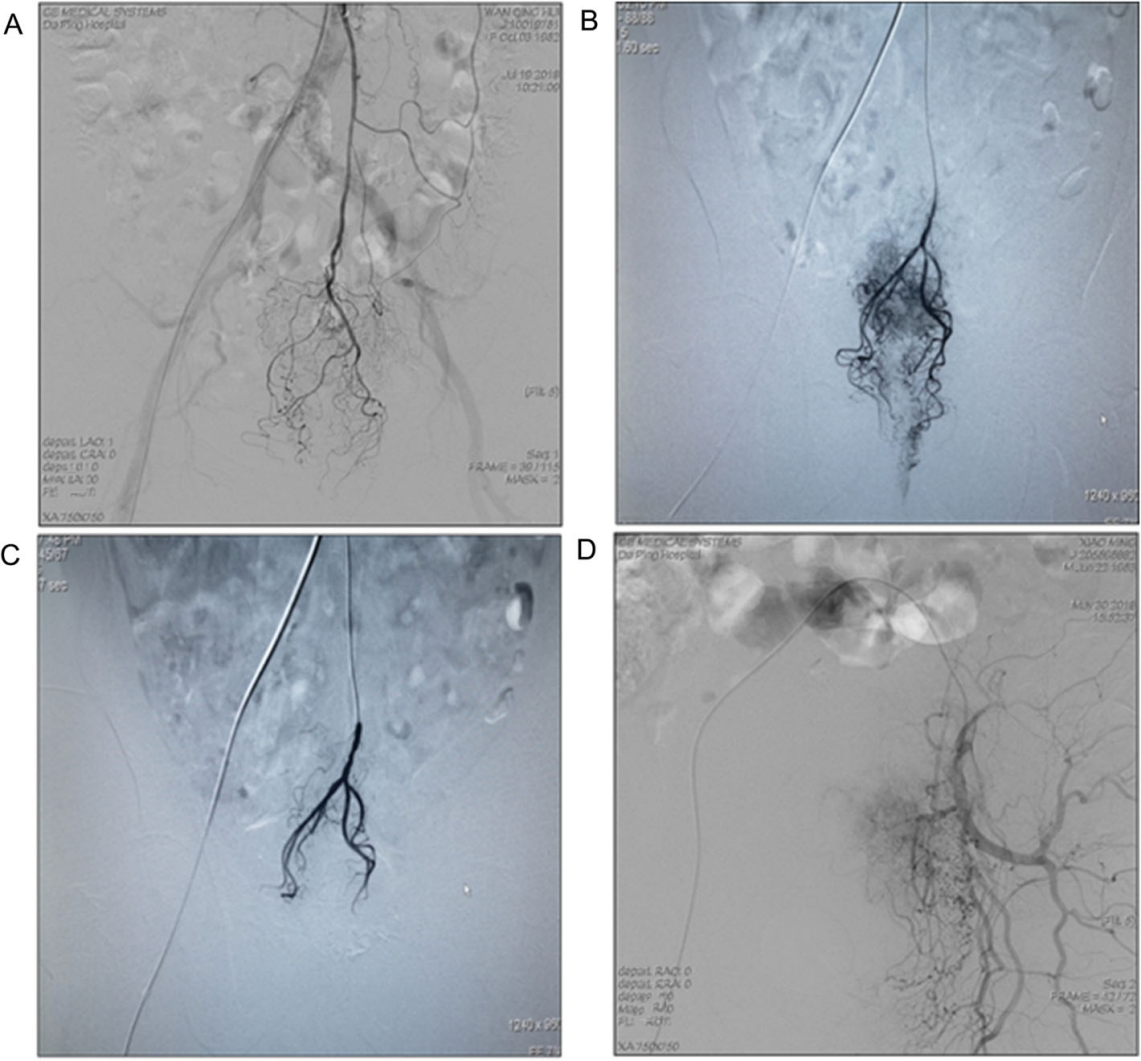
**a-d** shows the process of TRACE. **a** Frontal inferior mesenteric arteriogram shows a minimal hypervascular blush in the rectum. **b** Frontal selective superior rectal arteriogram shows a hypervascular blush within the rectum. **c** Frontal superior rectal arteriogram after TRACE shows no hypervascular blush with pruning of the arterial supply to the rectal tumor. **d** shows angiogram of the internal iliac artery

For TRACE in the NATRACE-CRT group, conventional femoral artery puncture and catheterization as well as angiography were conducted by Seldinger technique. Next, superselective catheterization of the superior rectal artery and inferior rectal artery was performed (to identify the primary tumor feeding arteries), in which the superselective catheters were inserted to each thickening and tortuous artery of the tumor lesions using a microcatheter. Subsequently, according to body-surface area (85 mg/m^2^), Oxaliplatin (Jiangsu Hengrui Medicine Co. Ltd., Nanjing, China) diluted to 50 ml normal saline was slowly infused (10 mins) via the transcatheter, after that the superior rectal artery was embolized using Gelatin Sponge Particle Embolic Agent (350-560 μm; Alicon Hangzhou, China) and 15 ml of Iodixanol, followed by angiography to confirm the embolism status. (In order to avoid tissue necrosis caused by simultaneous embolization of the superior rectal artery and the inferior rectal artery, we only selectively embolized the superior rectal artery.) The end-point of the embolization was the stagnation of blood flow in the feeding arteries conformed by angiography (Fig. 2). All operations were performed by the same interventional physician (He SY) as well.

In NATRACE-CRT group, S-1 (Taiho Pharmaceutical Company, Tokyo, Japan) was oral administered after arterial chemoembolization according to body-surface area (BSA), dose and times of administration as: BSA < 1.2 m^2^: 80 mg, 2/day; BSA = 1.2–1.5m^2^: 100 mg, 2/day; BSA > 1.5 m^2^: 120 mg/day, 2/day, 7d/week; on day 1 to day 28, and stop taking it for 14 days.

In the NA-CRT group, Patients received capecitabine during the radiotherapy, dose, and times of administration as: capecitabine (Shanghai Roche Pharmaceutical Co. Ltd., Shanghai, China) according to BSA (825 mg/m^2^), 2/day, 5d/week; on day 1 to day 35.

Both two groups received radiotherapy which delivered using a 4-field conformal coplanar technique (anteroposterior, posteroanterior, right lateral and left lateral fields) and 6–8 MV photon beams. A total dose of 45 Gy was given in 1.8 Gy fractions, five fractions per week, lasting for a total of 5 weeks.

Radical resection of rectal cancer was performed 4 weeks after completion of chemoradiotherapy. The principle of operation was total mesorectal excision, and sigmoid colostomy or ileostomy was routinely undertaken. Stoma closure was also carried out 6 months after surgery.

Although adjuvant treatment was not part a of the present study, however, following the National Comprehensive Cancer Network (NCCN) guidelines, adjuvant chemotherapy was recommended for all patients regardless of histology (i.e., in case of pathologic complete remission). Treatment was recommended to be followed for 4 to 6 months using mFOLFOX6 or CapeOx regimen.

Patients were followed up according to NCCN guidelines, history and physical every 3-6 months for 2 years, then every 6 months for a total of 5 years;CEA every 3 months for 2 years then every 6 months for a total 5 years;chest and abdominal/pelvic CT every 6 months for 2 years, then 12 months for a total 5 years.

### Evaluation of safety and efficacy

The assessment of clinical efficacy was performed by MRI according to the RECIST (Response Evaluation Criteria In Solid Tumors) 1.1 criteria [16]: complete remission (CR), disappearance of all target lesions; partial remission (PR), at least 30% decrease in the sum of diameters of target lesions; progression of disease (PD), at least 20% increase in the sum of diameters of target lesions or appearance of one or more new lesions; stable disease (SD), neither sufficient shrinkage to qualify PR nor sufficient increase to qualify PD; besides, objective response rate (ORR) was calculated by CR + PR.

**Fig. 3:**
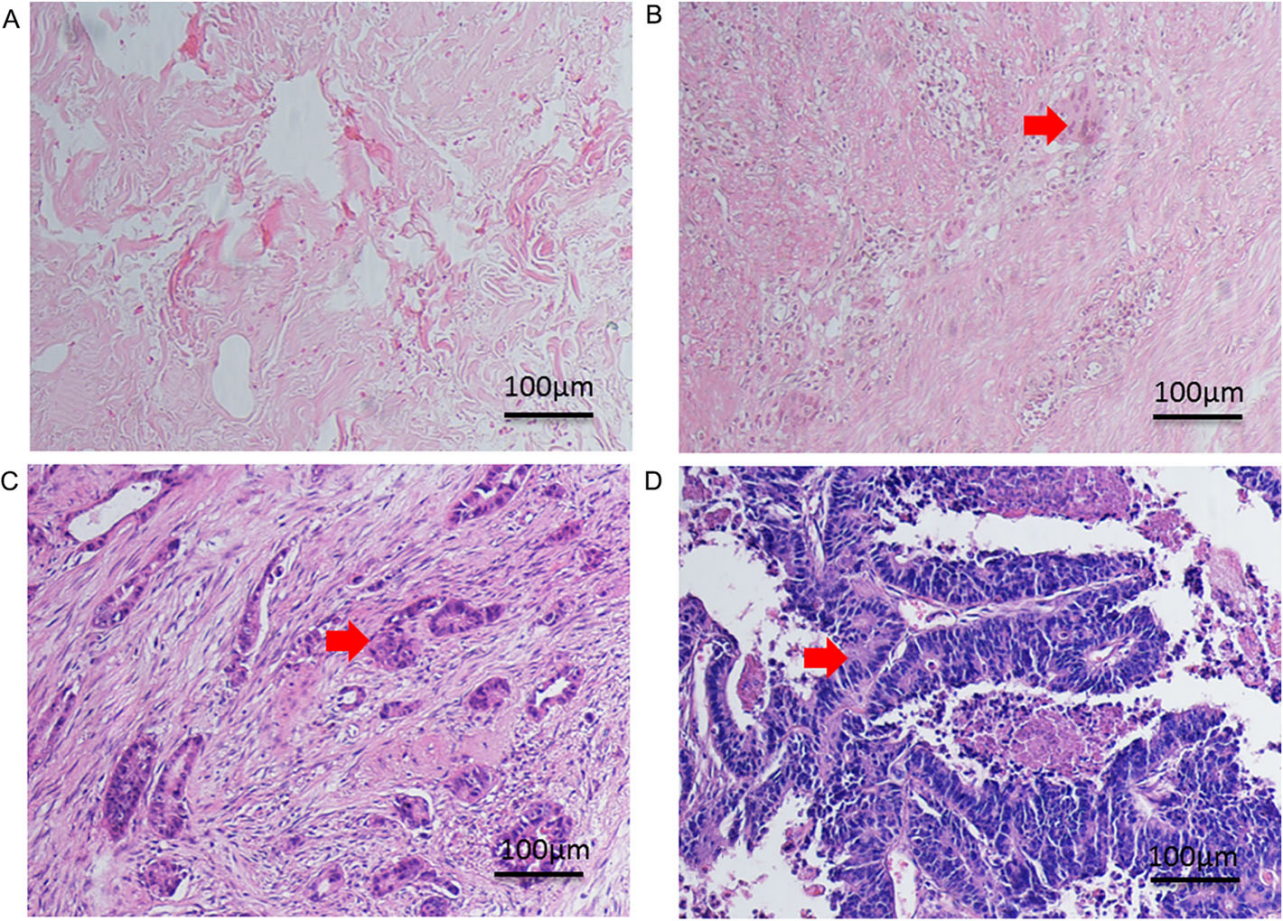
**a-d** shows different pathological findings of neoadjuvant therapy. HE staining of TRG0–3 grade (AJCC standard), respectively (**h**&**e**, original magnification = ×20, Bar scale = 100 μm), the arrows indicate residual tumor cells. **a** It shows TRG0 (pCR) complete remission without residual tumor cells; **b**. It displays TRG1 with only a single or few residual tumor cells; **c**. It shows less response to tumors with residual tumor cells; **d**. It shows TRG3 poor response to tumors, in which few or no tumor cells were killed. Arrows shows tumor cells

Tumor regression grade (TRG) was assessed according to the 7th edition of the American Joint Committee on Cancer (AJCC) standard [17]. TRG0 showed CR without residual tumor cells; TRG1 demonstrated a better response with only a single or very few residual tumor cells; TRG2 showed less response to tumors and still had residual tumor cells; TRG3 indicated a poor response to tumors, in which few or no tumor cells were killed (Fig. 3). The rectum was cut into transverse sections according to the Quirke procedure [18], and the TRG assessment was performed by two independent pathologists (DW, HL X).

Evaluation procedures, including physical examinations, measurement of vital signs, and complete blood count test, were performed weekly during neoadjuvant treatment. Toxicity was assessed and graded according to the Common Terminology Criteria for Adverse Events (CTCAE; version 4.0) issued by the National Cancer Institute.

### Study endpoints and statistical analysis

The primary endpoint is the pathological remission rate. The secondary endpoints include ORR and toxicity, as well as surgical complications and tumor downstaging.

Data were statistically analyzed by using SPSS 23.0 software (IBM, Armonk, NY, USA). The measured data were expressed as x ± s or mean (range), reflecting normal distribution of data,; besides, the independent sample *t*-test was used, demonstrating the non-normal distribution of data; the count data were expressed by the rate (%), and the Chi-square test was used as well. *P* < 0.05 was considered statistically significant. Cumulative survival probabilities were estimated using the Kaplan–Meier method, and differences between survival rates were tested for significance using the log-rank test.

## Results

### Patient characteristics

Patients’ characteristics are detailed in Table 1. The median age of the NATRACE-CRT group and NA-CRT group was 62 years (range, 20–75 years) and 57 (range, 32–75 years), respectively, and males accounted for 70.00 and 75.56% of the patients, respectively. There were 35 and 24 patients in stage of cT3, and the number of node positive cases was 36 and 27 in NATRACE-CRT group and NA-CRT group, respectively. In the NATRACE-CRT group, there were 15 cases in which tumors had a distance of more than 5 cm from the anal margin, and 17 cases were also found in the NA-CRT group. There was no significant difference in the demographic and disease characteristics of the two groups.

**Table 1:** Demographic characteristics of the patients

**Characteristic**	**NATRACE-CRT N**	**NA-CRT N**	***P*-value**
**Gender (%)**			
**Male**	35 (70.00)	34 (75.56)	0.647
**Female**	15 (30.00)	11 (24.44)	
**Age (years)**			
**Median**	62	57	0.063
**Range**	20–75	32–75	
**ECOG performance status (%)**			
**0**	43 (86.00)	37 (82.22)	0.784
**1**	7 (14.00)	8 (17.78)	
**BMI (kg/m**^**2**^**)**			
**Mean**	22.71 ± 3.07	24.18 ± 2.97	0.024
**Tumor length (mm)**			
**Mean**	4.15 ± 1.85	3.94 ± 1.31	0.293
**Distance from the AV (cm)**			
**Mean**	6.1 ± 2.63	5.73 ± 2.84	NA
**< 5**	15 (30.00)	17 (37.78)	
**≥ 5**	35 (70.00)	28 (62.22)	
**Clinical T stage**			
**T3**	35 (70.00)	24 (53.33)	0.138
**T4**	15 (30.00)	21 (46.67)	
**Clinical N stage**			
**N0**	14 (28.00)	18 (40.00)	0.455
**N1**	19 (38.00)	15 (33.33)	
**N2**	17 (34.00)	12 (26.67)	
**Baseline CEA level (ng/ml)**			
**Median**	2.88	3.29	0.516
**Range**	0.48–63.75	0.42–135.82	
**≤ 5.0**	35 (70.00)	28 (62.22)	
**> 5.0**	15 (30.00)	17 (37.78)	
**Histological grade**			
**Well differentiated**	6 (12.00)	6 (13.34)	0.979
**Moderately differentiated**	29 (58.00)	26 (57.78)	
**Poorly differentiated**	15 (30.00)	13 (28.88)	

Abbreviations: *ECOG* Eastern Cooperative Oncology Group, *BMI* body mass index, *AV* anal verge, *CEA* carcinoembryonic antigen

### Treatment exposure

One patient in the NATRACE-CRT group received irinotecan and bevacizumab for tumor progression during concurrent chemoradiotherapy. The rest of the patients were treated as planned.

The cumulative dose of radiotherapy in the NATRACE-CRT group and NA-CRT group was 41.28 ± 4.49 and 44.38 ± 5.35 Gy, respectively.

### Efficacy

#### Clinical response

Preoperative imaging examination revealed that 7 patients in the NATRACE-CRT group achieved CR, and 35 patients achieved PR, while 4 and 26 patients in the NA-CRT group achieved CR and PR, respectively. The ORR of the NATRACE-CRT group was 84% and that in the NA-CRT group was 66.67%, indicating that the ORR of the NATRACE-CRT group was higher than that in the NA-CRT group (*P* = 0.058). There was also no statistical significance between the two groups (Table 2).

**Table 2:** Objective Tumor Responses by Imaging

**Response**	**After NATRACE-CRT** (*n* = 50)	**After NA-CRT** (*n* = 45)
	No. of Patients (%)	No. of Patients (%)
**Complete response**	7 (14.00)	4 (8.89)
**Partial response**	35 (70.00)	26 (57.78)
**Stable disease**	7 (14.00)	15 (33.33)
**Progressive disease**	1 (2.00)	0 (0)
**Objective response rate, %**	42 (84.00)	30 (66.67)
**95% CI**	73.5–94.5%	52.3–81%
***P*value**	0.058	

In addition, 26 patients in the NATRACE-CRT group achieved a downstaging of T-stage, 26 patients achieved a downstaging of N-stage, and 31 of the 50 patients finally reached T or N downstaging. In the NA-CRT group, there were 17 cases with downstaging of T-stage and 16 cases with downstaging of N-stage, in which 22 patients reached T or N downstaging. The downstaging rates of the two groups were 62 and 48.89%, respectively (*P* = 0.22) (Table S1).

#### Surgery-related outcomes

In the NATRACE-CRT group, 50 patients underwent radical resection of rectal cancer, including 40 cases with low anterior resection, 9 cases with abdominal perineal resection, 1 case with total pelvic organ resection, and 50 cases with routine sigmoid colostomy. Only 1 patient underwent R1 resection and the remaining underwent R0 resection. All the patients achieved a negative circumferential margin (tumor distance > 1 mm). In the NA-CRT group, 30 cases were treated with low anterior resection, 13 cases with abdominal perineal resection, 2 cases with total pelvic organ resection, 45 cases with conventional sigmoid colostomy, and 45 cases achieved R0 resection and circumferential margin negative Table S2.

#### Pathological response

**Fig. 4:**
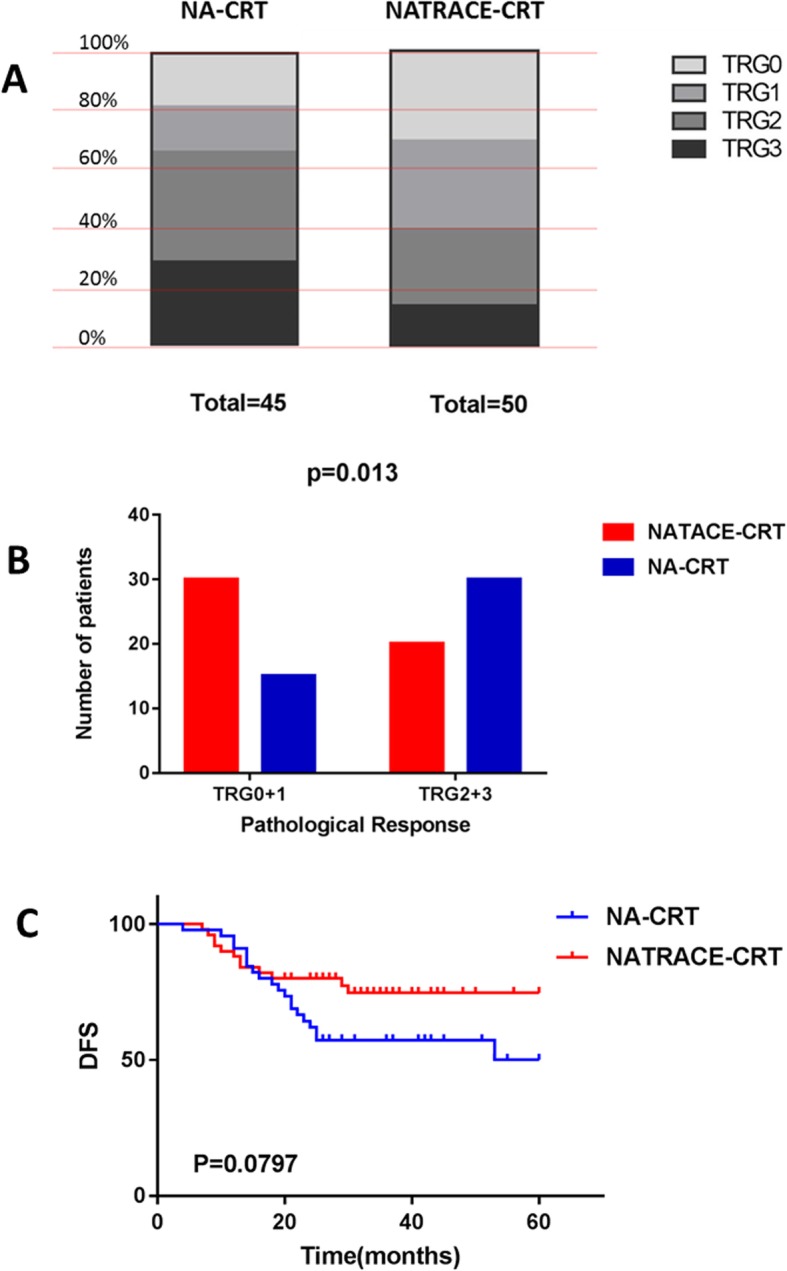
Pathological response rate and DFS. **a** It displays the tumor pathological regression graded in each group. There were 15 cases of TRG0 (pCR), 15 cases of TRG1, 13 cases of TRG2, and 7 cases of TRG3 in NATRACE-CRT group, while 8 cases of TRG0 (pCR), 7 cases of TRG1, 17 cases TRG2, and 13 cases of TRG3 were found in the NA-CRT group. **b** It shows that the TRG0 + 1 patient in NATRACE-CRT group had significantly more than patients in NA-CRT group. **c** Kaplan–Meier survival analysis of DFS in two groups, *p* = 0.0797

Tumor regression grade was assessed according to the AJCC criteria, of 95 cases of surgical resection specimens, there were 15 cases of TRG0 (pCR), 15 cases of TRG1, 13 cases of TRG2, and 7 cases of TRG3 in NATRACE-CRT group, while 8 cases of TRG0 (pCR), 7 cases of TRG1, 17 cases TRG2, and 13 cases of TRG3 were found in the NA-CRT group (Fig. 4a). The pCR rate in the NATRACE-CRT group was 30% (95% confidence interval (CI): 16.8–43.2) and 17.78% (95% CI: 6.2–29.4) in the NA-CRT group (*P* = 0.231), respectively. Although there was no statistical difference in pCR rate between the two groups, however, the TRG0 + 1 rate in the two groups was 60% (95% CI: 45.9–74.1) and 33.33% (95% CI: 19–47.7), respectively, which showed a remarkable difference (*P* = 0.013) (Fig. 4b). This result demonstrated that tumor regression rate of the NATRACE-CRT group was significantly higher than that of the NA-CRT group.

The follow-up time of NATRACE-CRT group was 7–60 months, and the median follow-up time was 33 months, the NA-CRT group was 4–60 months, and the median follow-up time was 27 months. We analyzed the DFS of the two groups. There was no statistical difference between the two groups (Fig. 4c). The median DFS of the two groups was not reached. The DFS rates of the two groups in 1 year and 3 years were 88% vs.92, 76% vs.58%, *P* = 0.0797.

### Toxicity

#### TRACE and Chemoradiotherapy-related toxicity

The occurrence of adverse events in the NATRACE-CRT group and the NA-CRT group was similar; for example, adverse events of grade 3–4, including leukopenia (6, 4.44%) and radiation proctitis (2, 2.22%); adverse events of grade 1–2, mainly including loss of appetite (32, 40%), followed by fatigue (24%, 24.44), diarrhea (24, 20%), and anemia (16, 13.33%). The incidence of toxicity and side effects of grade 3–4 was 14% in NATRACE-CRT group and 13.33% in NA-CRT group, respectively. Moreover, there were 15 cases of radiation proctitis in the NATRACE-CRT group and 8 cases in the NA-CRT group. There were no statistically significant differences in adverse events between the two groups. The details of adverse events are shown in Table 3.

**Table 3:** Adverse events during chemoradiotherapy (*n* = 95)

	**G1 + G2 (%)**			**G3 + G4 (%)**		
	**NATRACE-CRT**	**NA-CRT**	***P*-value**	**NATRACE-CRT**	**NA-CRT**	***P*-value**
**Leukopenia**	7 (14.00)	3 (6.67)	0.324	3 (6.00)	2 (4.44)	0.735
**Neutropenia**	3 (6.00)	1 (2.22)	0.619	1 (2.00)	1 (2.22)	0.941
**Anemia**	8 (16.00)	6 (13.33)	0.778			
**Thrombocytopenia**	3 (6.00)	1 (2.22)	0.619	1 (2.00)	1 (2.22)	0.941
**Febrile neutropenia**	0	1 (2.22)	NA		1 (2.22)	NA
**AST**	0	1 (2.22)	NA			
**ALT**	0	1 (2.22)	NA			
**Total bilirubin**	1 (2.00)	1 (2.22)	0.941			
**Nausea**	15 (30.00)	11 (24.44)	0.647			
**Vomiting**	4 (8.00)	3 (6.67)	0.804			
**Fatigue**	12 (24.00)	11 (24.44)	0.960			
**Diarrhea**	12 (24.00)	9 (20.00)	0.805			
**Appetite loss**	16 (32.00)	18 (40.00)	0.521			
**Hand–foot syndrome**	1 (2.00)	0	NA			
**Radiation proctitis**	15 (30.00)	12 (26.67)	0.719	1 (2.00)	2 (4.44)	0.496

Abbreviations: *AST* aspartate aminotransferase, *ALT* alanine aminotransferase. National Cancer Institute Common Toxicity Criteria, version 4.0

#### Surgery-related toxicity

Postoperative complications in the NATRACE-CRT group included incision infection (1/50), pelvic infection (2/50), and anastomotic leakage (1/50). The complications of the NA-CRT group included pelvic infection (1/45), anastomotic leakage (1/45), and intestinal obstruction (1/45). There was no significant difference in operative methods and complications between the two groups. The details of surgery and surgical complications are shown in Table S2.

## Discussion

TACE has been extensively used in the treatment of advanced hepatocellular carcinoma, possessing a satisfactory effect on local control of tumors, while it has rarely been reported in other types of tumors. Adding oxaliplatin to the regimen of adjuvant chemotherapy based on 5-FU has improved DFS and OS in colon cancer [19], Therefore, several large randomized phases III trials tested the efficacy of adding oxaliplatin to the multimodal neoadjuvant treatment for LARC. The CAO/ARO/AIO-04 [20] study is the only one with positive results at present. STAR-01 [21], ACCORD12 [22], and NSABP-R04 [23] studies all suggested that oxaliplatin did not possess survival benefits. The interim analysis of FOWARC [24] revealed that oxaliplatin could improve the pCR rate, while the final results did not present survival benefits. In addition, several meta-analyses also suggested that the addition of oxaliplatin to neoadjuvant therapy could improve the rate of pCR and reduce the rate of distant metastasis [25, 26].

Several studies have found that pathological regression was correlated with local control of tumors, distant metastasis, DFS, and OS [10, 27, 28], thus we selected pathological regression as a short-time curative effect, and found that TRACE with oxaliplatin can improve the rates of pCR and TRG0 + 1, which would be comparable to FOLFOX in 6 cycles before surgery [24], and higher than that of oxaliplatin intravenous administration (14–19.2%) [20, 21, 23, 29, 30].

A series of Japan studies have found that S-1 adjuvant chemotherapy has similar efficacy to Tegafur. In addition, when S-1 is used as a neoadjuvant therapy regimen, it can improve the pCR rate [14, 15]. A meta-analysis compared the safety and efficacy of S1 or 5-FU-based chemotherapy for advanced colorectal cancer [31], which showed that no difference in OS, PFS, and ORR between the two groups, while there was lower incidence in grade 3–4 neutropenia, nausea, and vomiting with S-1 treatment. Our study found that S-1 single drug chemotherapy combined with TARCE can significantly improve the rate of pathological remission.

The adverse events of NATRACE-CRT were well tolerated. No relevant treatment-related deaths were found. The most common adverse events in grade1–2 were loss of appetite and fatigue, and diarrhea symptoms. There were no obvious neurological symptoms and skin toxicity and mucositis caused by oxaliplatin. Radiation proctitis occurred in 15 patients, while they were mostly in grade 1–2, similar to that in the NA-CRT group. In NATRACE-CRT group, the dose of oxaliplatin was 85 mg/m^2^, one time, which were 85 mg/m^2^ × 6 times in the FOWARC study, and 60 mg/m^2^ × 6 times in the STAR-01 study. The cumulative dose of oxaliplatin was notably lower than that in previous studies, which might be related to the relatively low incidence of toxic side effects in the present study.

The NATRACE-CRT group did not increase the complications. In this study, patients in the NATRACE-CRT group received surgery 4 weeks after the completion of chemoradiation. Previous studies have shown that the maximum pCR rate can be achieved by 8–12 weeks of operation interval [32–34]. Therefore, we speculate that on the basis of this study, if the operation interval is prolonged, a higher pCR rate may be obtained. The limitations of this study were small sample size and insufficient follow-up.

## Conclusion

TRACE with oxaliplatin plus S-1 concurrent chemoradiotherapy as a neoadjuvant therapy provided better pathological remission rate versus standard treatment with a similar safety profile.

## Supplementary information

**Additional file 1: Table S1.1.** Pathologic Response-T stage. **Table S1.2.** Pathologic Response-N stage. **Table S2.** Surgical Procedures and complications.

## Data Availability

Not applicable.

## Abbreviations

TRACE: Transcatheter rectal arterial chemoembolization
TRG: Tumor regression grade
ORR: Objective response rate
CRT: Chemoradiotherapy
TME: Total mesorectal excision
LARC: Locally advanced rectal cancer
pCR: Pathologic complete remission
DFS: Disease-free survival
OS: Overall survival
