# Supplementary material for: Transcatheter rectal arterial chemoembolization with oxaliplatin plus S-1 concurrent chemoradiotherapy can improve the pathological remission rate in locally advanced rectal cancer: a comparative study
Source: Radiat Oncol. 2020 May 6;15:94. doi: 10.1186/s13014-020-01540-4 (PMC7201605; doi:10.1186/s13014-020-01540-4)
Supplement: Supplementary file 1 — Additional file 1: Table S1.1. Pathologic Response-T stage. Table S1.2. Pathologic Response-N stage. Table S2. Surgical Procedures and complications. [file 13014_2020_1540_MOESM1_ESM.doc]

Table S1.1 Pathologic Response-T stage

| **Baseline**  **Staging** | **Pathologic Staging** | | | | | | | | | |
| --- | --- | --- | --- | --- | --- | --- | --- | --- | --- | --- |
|  | pT0 | | pT1 | | pT2 | | pT3 | | pT4 | |
|  | NA-CRT | NATACE-CRT | NA-CRT | NATACE-CRT | NA-CRT | NATACE-CRT | NA-CRT | NATACE-CRT | NA-CRT | NATACE-CRT |
| **cT3** | 6 | 9 | 0 | 0 | 2 | 7 | 9 | 15 | 7 | 4 |
| **cT4** | 2 | 6 | 0 | 0 | 1 | 0 | 6 | 4 | 12 | 5 |

Table S1.2 Pathologic Response-N stage

| **Baseline**  **Staging** | **Pathologic Staging** | | | | | |
| --- | --- | --- | --- | --- | --- | --- |
|  | N0 | | N1 | | N2 | |
|  | NA-CRT | NATACE-CRT | NA-CRT | NATACE-CRT | NA-CRT | NATACE-CRT |
| **N0** | 13 | 11 | 4 | 5 | 1 | 0 |
| **N1** | 7 | 12 | 6 | 5 | 2 | 0 |
| **N2** | 9 | 10 | 0 | 4 | 3 | 3 |

Table S2. Surgical Procedures and complications

| **Surgical approach** | **NATACE-CRT (n=50)** | | **NA-CRT (n=45)** | | **P-value** | |
| --- | --- | --- | --- | --- | --- | --- |
|  | n | % | n | % |  |  |
| **Open** | 5 | (10.00) | 3 | (6.67) | | 0.734 |
| **Laparoscopic** | 43 | (86.00) | 41 | (91.11) | |  |
| **da Vinci Surgical System** | 2 | (4.00) | 1 | (2.22) | |  |
| **Operative procedures** |  | | | | |  |
| **Low anterior resection** | 40 | (78.72) | 30 | (61.11) | | 0.328 |
| **Abdominoperineal resection** | 9 | (19.15) | 13 | (33.33) | |  |
| **Total pelvic exenteration** | 1 | (2.13) | 2 | (5.56) | |  |
| **Lateral lymph node dissection** | 50 | (100) | 45 | (100) | | NA |
| **surgery complications** |  |  |  |  | | 0.718 |
| **Wound sepsis** | 1 | (2.13) |  |  | |  |
| **Pelvic sepsis** | 2 | (4.26) | 1 | (2.22) | |  |
| **Anastomotic leakage** | 1 | (2.13) | 1 | (2.22) | |  |
| **Ileus** |  |  | 1 | (2.22) | |  |
| **Residual tumor classification** |  | | | | | NA |
| **R0** | 49 | (98.00) | 44 | (97.78) | |  |
| **R1** | 1 | (2.00) | 1 | (2.22) | |  |
| **R2** | 0 |  | 0 |  | |  |
